# Endoscopic Removal of Superglue From the Urethra: A Case Report and Review of the Literature

**DOI:** 10.1155/criu/2780056

**Published:** 2025-04-18

**Authors:** Marcus Derigs, Aristeidis Zacharis, Cem Aksoy, Subhajit Mandal, Johannes Huber, Christer Groeben

**Affiliations:** ^1^Department of Urology, Philipps-University Marburg, Marburg, Hesse, Germany; ^2^Department of Urology, Oberlausitz-Kliniken, Bautzen, Saxony, Germany; ^3^Department of Urology, Catholic Hospital, Sankt Johann Nepomuk, Erfurt, Thuringia, Germany; ^4^Department of Urology, University Hospital Heidelberg, National Center for Tumor Diseases (NCT), Heidelberg, Baden-Württemberg, Germany

**Keywords:** autoerotic manipulation, endoscopic removal, literature review, rigid cystoscopy, superglue, urethra, urethral obstruction, urethral polyembolokoilamania, urinary retention

## Abstract

**Background:** The self-insertion of superglue into the male urethra is an uncommon and challenging cause of urethral obstruction, with only five reported cases to date. These cases demonstrate diverse clinical presentations and treatment approaches. This report presents the sixth documented case, reviews the existing literature, and proposes a structured treatment approach for superglue-induced urethral obstruction.

**Case Presentation:** A 51-year-old male instilled superglue into his urethra to secure a pintail comb inserted for self-stimulation. Upon forcefully removing the comb, hardened superglue fragments remained stuck in the urethra, causing urinary retention. Endoscopic extraction using rigid cystoscopy and forceps under local anesthesia successfully removed all fragments from the penile urethra and bladder.

**Conclusion:** Early endoscopic extraction using rigid cystoscopy under local anesthesia represents a safe and effective treatment option for nonadherent superglue fragments in the urethra.

## 1. Introduction

The insertion of a foreign object into the male urethra is a rare but serious urological emergency, often resulting in urinary obstruction, urethral trauma, and long-term complications. Approximately 1350 cases are reported annually in the United States [1]. These incidents are typically self-inflicted and may result from autoerotic stimulation, curiosity, intoxication, or an underlying psychiatric condition [2]. A wide range of foreign bodies has been documented, including fishhooks, metal rods, bones, screws, pellets, and light bulbs [3]. Superglue instillation into the urethra is particularly uncommon, with only five documented cases in the medical literature to date [4–8]. The rapid polymerization of cyanoacrylate adhesives (i.e., superglue) upon contact with moisture results in immediate solidification, creating a challenging scenario for removal due to the high risk of strong adhesion and mucosal injury.

This report describes the sixth documented case, demonstrating the feasibility of early rigid cystoscopy under local anesthesia. Additionally, a review of the literature is provided to guide clinical decision-making and propose a structured treatment framework for managing superglue-induced urethral obstruction.

## 2. Case Presentation

A 51-year-old male presented to the emergency department after instilling superglue into his urethra to affix the handle of a pintail comb for self-stimulation. His medical history revealed a 20-year pattern of urethral foreign body insertion, with the most recent involving a hex wrench 3 years earlier. These episodes were consistently associated with substance use, including alcohol and amphetamines, and had previously necessitated endoscopic procedures for foreign body removal and urethral strictures. Despite multiple referrals for alcohol deaddiction therapy, the patient declined treatment, citing personal reasons. His medical history was notable for alcoholic hepatopathy and chronic gastritis. He denied recent urinary symptoms, allergies, psychiatric disorders, or a relevant family history.

Before the current incident, the patient consumed a bottle of whiskey and approximately 1 g of amphetamine. He inserted the pintail comb handle into his urethra for several hours and subsequently instilled two tubes (3 mL each) of superglue (cyanoacrylate; Pattex; Henkel, Dusseldorf, Germany) to secure it in place. After 4 h, he forcefully removed the comb. Over the next 7 h, he developed worsening obstructive voiding symptoms and sought emergency medical care.

Physical examination revealed a palpable object approximately 3 cm deep into the penile urethra, mild glans swelling, and no visible superglue at the meatus. The patient was able to void only a few drops of urine, and ultrasonography revealed a postvoid residual volume of 200 mL. He also presented with tachyarrhythmia (220 bpm) and was initially admitted to the chest pain unit for cardiac monitoring. Following spontaneous improvement of the tachycardia and increasing lower abdominal pain due to urinary retention, an interdisciplinary decision was made to prioritize urological treatment, with plans for further cardiologic evaluation afterward.

**Figure 1:**
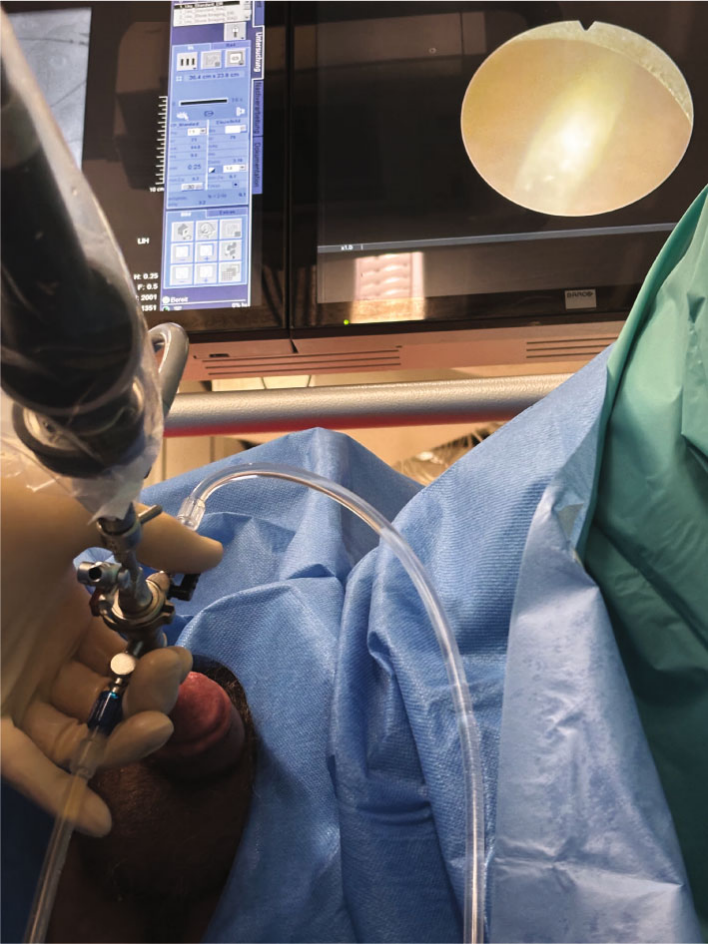
Rigid urethrocystoscopy showing a large fragment of hardened superglue in the bladder (upper right corner). Prior to this, smaller fragments were successfully removed from the urethra.

**Figure 2:**
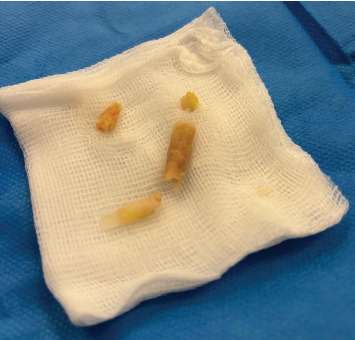
Four fragments (2.0, 1.2, 0.8, and 0.5 cm in length, each up to 0.5 cm in width) were evacuated from the penile urethra and bladder.

**Figure 3:**
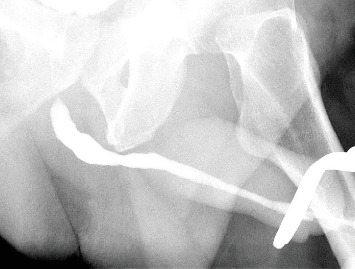
Retrograde urethrogram 10 days after endoscopic removal of superglue fragments showing no urethral damage.

The patient consented to urethrocystoscopy for diagnostic evaluation and possible fragment removal, with the alternative of suprapubic catheter placement if removal was unsuccessful. Three hours post-presentation, a rigid 19.5 Fr cystoscope (Figure 1) was introduced under local anesthesia (10 mL lidocaine gel), supplemented by intravenous metamizole (1 g) for pain control. Three hardened superglue fragments (up to 1.5 cm each) were identified in the midpenile urethra and extracted separately using a stent removal forceps. The fragments, while obstructing the urethral lumen, were nonadherent to the mucosa, allowing straightforward removal. Additionally, a 2-cm fragment was visualized in the bladder and removed without difficulty (Figure 2). Minor mucosal lacerations were noted, but no other complications occurred. A three-way Foley catheter was inserted, draining clear urine, and the procedure was completed. The patient reported minimal discomfort and complete resolution of symptoms postoperatively. He underwent successful electrical cardioversion the following day and was discharged with the urinary catheter in place. At the 10-day follow-up, the patient had an uneventful recovery. The catheter was removed painlessly, and a retrograde urethrogram confirmed no residual urethral damage (Figure 3). He was able to void spontaneously, pain-free, and without postvoid residual urine. He was counseled on the risks of recurrent foreign body insertion and was strongly encouraged to seek psychiatric treatment to address his substance use disorder. Given his history of repeated urethral instrumentation for autoeroticism, he was suggested he could use a lubricated, single-use catheter as a safer nontraumatic alternative.

At the 6-month follow-up, he remained asymptomatic, with no urinary symptoms or postvoid residual. He reported abstinence from major drug use, aside from alcohol consumption, and denied further autoerotic behaviors. However, he had not sought psychiatric treatment or utilized self-catheterization. Yearly urological follow-ups were recommended to monitor for potential long-term complications.

## 3. Discussion

The insertion of foreign objects into the urethra is a rare but significant urologic emergency [1]. Among the various reported cases, superglue instillation is particularly uncommon, with only five prior cases documented in the medical literature [4–8]. Unlike other foreign objects that can often be retrieved with relative ease, superglue solidifies within the urethra and may adhere to its walls, making extraction more complex and increasing the risk of mucosal injury.

A review of previously reported cases provides valuable insights into the varying clinical scenarios and management strategies (Table 1). Treatment approaches have ranged from simple extraction to more invasive procedures such as urethrotomy [4–8].

Turner managed the first documented case by successfully removing a 7-cm fragment from the penile urethra through meatal extraction and a loosely adherent 2-cm fragment from the posterior urethra via endoscopic removal using biopsy forceps under general anesthesia [4].

Heberling et al. faced complications with firmly adherent superglue fragments. Initial conservative management failed, and a subsequent endoscopic attempt 19 days postinstillation was unsuccessful due to firm adherence to the urethral wall. Complete removal ultimately required an external urethrotomy [5].

Khoo et al. treated a patient with superficial superglue application to the glans and meatus by soaking the foreskin with warm, soapy water and manually removing the fragments under general anesthesia [6].

Young et al. encountered a unique challenge in a patient who had instilled 20 mL of superglue, far exceeding the typical 1–4 mL found in standard superglue tubes. The initial endoscopic attempt failed due to meatal obstruction. Treatment was delayed for 6 months due to the patient's noncompliance, which was only possible because he retained sufficient voiding ability. Treatment involved urethral dilation and attempted laser fragmentation (0.6 J), both of which proved ineffective. The eventual removal of three fragments was accomplished using a rigid cystoscope and cold cup biopsy forceps under general anesthesia [7].

Khalili Fomeshi et al. highlighted the benefits of extensive urethral irrigation in a case where complete meatal obstruction prevented endoscopic access. Following a glans incision, 3 L of saline was irrigated through the urethra over 15 min, which facilitated the removal of a 10-cm urethral fragment using forceps via the meatus [8].

Follow-up data from most cases reported no complications at 6 weeks, though longer-term outcomes remain unclear [5, 7, 8].

In the present case, endoscopic treatment under local anesthesia was performed 3 h after presentation and successfully removed all superglue fragments from the penile urethra and bladder. This approach avoided the need for general anesthesia, which was required in prior cases [4, 7]. The patient's tachyarrhythmia posed additional risks for general anesthesia, and his preference for a prompt resolution further supported the choice of local anesthesia. Nevertheless, a contingency plan for conversion to general anesthesia was in place in case of patient discomfort or technical difficulties. Rigid cystoscopy was selected over flexible cystoscopy for its superior visualization, capacity for larger irrigation volumes, and compatibility with a more robust stent removal forceps. Despite its association with greater discomfort and higher complication rates, the advantages of rigid cystoscopy were pivotal in achieving a successful outcome in this case. To mitigate the risk of urethral stricture by continued self-stimulation with foreign objects, self-catheterization was recommended based on our clinical judgement, as no common recommendations exist regarding this issue. However, given the potential to reinforce autoerotic behavior, its necessity was carefully weighed. At the 6-month follow-up, the patient had not adopted this practice.

The collective experiences from these cases highlighted the importance of tailoring treatment to the specific clinical scenario, considering factors such as the location, adherence, and volume of the superglue. Based on our review of prior cases and our experience, we propose a preliminary treatment framework (Table 2).

For cases where superglue is localized to the foreskin, glans, or meatus, initial management may involve soaking the foreskin in warm, soapy water for 10 min to soften and loosen the adhesive [6]. Visible fragments can then be manually peeled off. Given the region's rich sensory innervation, general anesthesia is advised to minimize discomfort. Endoscopy should follow to rule out deeper fragments within the urethra.

For intraurethral fragments, the management approach depends on their visibility and size. Fragments visible and accessible via the meatus can be removed using forceps. Larger fragments that cannot be extracted through the meatus may need a meatal incision for removal [8]. Nonvisible fragments require prompt rigid cystoscopy to assess their adherence and enable possible simultaneous removal, a procedure that necessitates adequate anesthesia. While local anesthesia may be appropriate for some cases, it is more commonly associated with discomfort, technical limitations, and longer procedural times, which may lead to greater irrigation volumes and the use of larger instruments. For these reasons, general or regional anesthesia is typically recommended. If adequate anesthesia support is not available or if the patient is not deemed fit for general anesthesia, a suprapubic catheter may be placed instead. Although general or regional anesthesia remains the standard, local anesthesia may still represent a feasible alternative—particularly when guided by individual perioperative risk profiles, patient preference, or limited anesthesia availability.

For cases involving complete meatal obstruction or firmly adherent fragments, additional interventions such as saline irrigation may facilitate fragment mobilization, allowing for endoscopic retrieval using forceps [8]. When these measures fail, external urethrotomy should be considered as a definitive approach [5].

Several important general considerations should guide the management of these cases. Regarding the timing of intervention, prompt action is generally recommended—particularly in symptomatic patients—to prevent further complications, such as infection, urethral strictures, and worsening obstruction. In contrast, in asymptomatic patients with low postvoid residual volumes, delayed management may be possible [7]. Importantly, even in reported cases of complete urethral obstruction with adherent superglue, suprapubic catheter placement was not required despite unsuccessful endoscopic removal [5, 7]. Nonetheless, when endoscopic extraction is not feasible and urinary retention occurs, suprapubic diversion remains a valid option. Supportive measures like increased fluid intake or laser fragmentation have shown no efficacy in superglue removal and should not be relied upon [5, 7]. Although solvents such as acetone have been effective in dissolving superglue in other anatomical sites [9], their application in the urethra is discouraged due to mucosal toxicity observed in animal models [10]. In cases where urethral injury is suspected, intraoperative or postoperative retrograde urethrograms can help in assessing the extent of damage. Placement of a transurethral catheter for several days is recommended to promote mucosal healing and ensure urinary drainage.

Following successful acute treatment, emphasis should be placed on addressing the underlying factors contributing to autoerotic behavior, including psychiatric conditions and substance abuse. Psychiatric consultation and addiction counseling should be strongly encouraged to mitigate recurrence.

### 3.1. Take-Away Lessons

This case demonstrates that early, endoscopic management with rigid cystoscopy is a safe, fast, and effective approach for the removal of nonadherent intraurethral fragments. It expands the available treatment options for superglue-induced urethral obstruction, which should be individualized based on the specific characteristics of the superglue fragments, including their location, degree of adherence, and extent.

## Figures and Tables

**Table 1:** Clinicopathological features of cases with urethral superglue instillation reported in the literature.

**Case**	**Author (Y)**	**Country**	**Age (year)/sex**	**Symptoms**	**Amount of superglue instilled**	**Time until presentation**	**Location of superglue obstruction**	**Management**
1 [4]	Turner (1990)	England	22/M	None	Not mentioned	Same day	Anterior (penile) and posterior urethra	- Extraction of a 7 cm anterior urethral fragment via meatus under anesthesia - Endoscopic removal of 2 cm posterior urethral fragment using biopsy forceps under general anesthesia - No postoperative catheter

2 [5]	Heberling (2016)	Germany	18/M	Dysuria and urinary flow reduction	Not mentioned	Same day	Anterior (penile) urethra	- Increased fluid intake, prophylactic antibiotics for 10 days - Endoscopic removal failed due to firm adherence to urethral wall, 19 days postinstillation - External urethrotomy for removal of all fragments - Postoperative catheter for 10 days

3 [6]	Khoo (2016)	UK	19/M	Urinary retention	One tube	Next day	Glans, meatus	- Soaking of the foreskin in warm, soapy water for 10 min - Manual removal of superficial glue deposits from glans and meatus under general anesthesia - Postoperative catheter for 1 day

4 [7]	Young (2016)	UK	39/M	5 days later: none 4 months later: dysuria, urinary hesitancy, and swollen glans	20 mL	5 days	Meatus, anterior (bulbar) urethra	- Initial endoscopic removal attempt failed due to meatal obstruction, 5 days postinstillation -6 months later: endoscopy and dilation of urethra to 24 Fr under general anesthesia: - Attempts at laser fragmentation (0.6 J) and grasping with stent removal forceps failed - Complete removal of three fragments achieved with cold cup biopsy forceps - No postoperative catheter

5 [8]	Khalili Fomeshi (2020)	Iran	41/M	4 days later: dysuria and oliguria 2 weeks later: urinary retention	Not mentioned	14 days	Meatus, anterior (penile) Urethra	- Endoscopic removal failed due to meatal obstruction - Glans incision and extensive saline irrigation of the urethra (3 L, ~15 min) under general anesthesia: - Removal of adherent 10 cm urethral fragment via meatus with a forceps, without endoscopy - Postoperative catheter for 10 days - Postoperative Amikacin 500 mg BD and cefazolin 1 g QID for 5 days

**Table 2:** Treatment recommendations for different scenarios of superglue-induced urinary obstruction.

**Location/characteristics of superglue**	**Treatment recommendation**
Foreskin and/or glans	1. Soak the foreskin in warm, soapy water to soften and loosen the superglue for > 10 min. 2. Manually remove (“peel off”) any visible fragments. 3. Perform endoscopy to rule out intraurethral fragments.

Meatus, urethra and/or bladder	1. Remove visible fragments with forceps via meatus. 2. Perform meatal incision for larger fragments. 3. Conduct early rigid cystoscopy for nonvisible fragments to assess adherence and perform simultaneous removal if possible. - Proceed with general anesthesia if local anesthesia proves insufficient.

Meatal obstruction or firm adherence to urethral wall	1. Irrigate the urethra with saline to loosen adherent fragments. 2. Remove fragments using endoscopy with forceps. 3. Consider external urethrotomy if irrigation fails.

## Data Availability

The datasets used and/or analyzed during the current study are available from the corresponding author on reasonable request. All data generated or analyzed during this study are included in this published article.
